# Using a tailored health information technology- driven intervention to improve health literacy and medication adherence in a Pakistani population with vascular disease (Talking Rx) – study protocol for a randomized controlled trial

**DOI:** 10.1186/s13063-016-1244-1

**Published:** 2016-03-05

**Authors:** Ayeesha Kamran Kamal, Abdul Muqeet, Kashfa Farhat, Wardah Khalid, Anum Jamil, Ambreen Gowani, Aliya Amin Muhammad, Fabiha Zaidi, Danyal Khan, Touseef Elahi, Shahrukh Sharif, Sibtain Raz, Taha Zafar, Syedah Saira Bokhari, Nasir Rahman, Fateh Ali Tipoo Sultan, Saleem Sayani, Salim S. Virani

**Affiliations:** The International Cerebrovascular Translational Clinical Research Training Program, Aga Khan University, Stadium Road, 74800 Karachi, Pakistan; eHealth Innovation, Aga Khan Development Network eHealth Resource Centre, Karachi, Pakistan; Talking Rx Study, Stroke Service, Aga Khan University Hospital, Karachi, Pakistan; Stroke Service, The International Cerebrovascular Translational Clinical Research Training Program, Aga Khan University, Karachi, Pakistan; Stroke Service, Department of Medicine, The International Cerebrovascular Translational Clinical Research Training Program, Aga Khan University, Karachi, Pakistan; Program Software Development and Integration, Aga Khan Development Network eHealth Resource Centre, Karachi, Pakistan; Aga Khan Development Network eHealth Resource Centre, Pakistan and Stroke Service, Aga Khan University Hospital, Karachi, Pakistan; Aga Khan Development Network, eHealth Resource Centre, Karachi, Pakistan; eOcean, For Talking Rx Interactive Voice Response Systems Solutions, Karachi, Pakistan; Department of Medicine, Aga Khan University Hospital, Karachi, Pakistan; Section of Cardiovascular Research, Department of Medicine, Baylor College of Medicine, and Staff Cardiologist, Michael E. DeBakey Veterans Affairs Medical Center, Houston, TX USA

**Keywords:** Vascular disease, Medication adherence, Health literacy, Talking prescription, Information and communication technology, Prevention, Non-communicable disease, Prevention, Lower and middle income countries

## Abstract

**Background:**

Vascular disease, manifesting as myocardial infarction and stroke, is a major cause of morbidity and mortality, especially in low- and middle-income countries. Current estimates are that only one in six patients have good adherence to medications and very few have sufficient health literacy. Our aim is to explore the effectiveness and acceptability of Prescription Interactive Voice Response (IVR) Talking Prescriptions (Talking Rx) and SMS reminders in increasing medication adherence and health literacy in Pakistani patients with vascular disease.

**Methods:**

This is a randomized, controlled, single center trial. Adult participants, with access to a cell phone and a history of vascular disease, taking multiple risk-modifying medications (inclusive of anti-platelets and statins) will be selected from cerebrovascular and cardiovascular clinics. They will be randomized in a 1:1 ratio via a block design to the intervention or the control arm with both groups having access to a helpline number to address their queries in addition to standard of care as per institutional guidelines. Participants in the intervention group will also have access to Interactive Voice Response (IVR) technology tailored to their respective prescriptions in the native language (Urdu) and will have the ability to hear information about their medication dosage, correct use, side effects, mechanism of action and how and why they should use their medication, as many times as they like. Participants in the intervention arm will also receive scheduled SMS messages reminding them to take their medications. The primary outcome measure will be the comparison of the difference in adherence to anti-platelet and statin medication between baseline and at 3-month follow-up in each group measured by the Morisky Medication Adherence Scale. To ascertain the impact of our intervention on health literacy, we will also compare a local content-validated and modified version of Test of Health Literacy in Adults (TOFHLA) between the intervention and the control arm.

We estimate that a sample size of 86 participants in each arm will be able to detect a difference of 1 point on the MMAS with a power of 90 % and significance level of 5 %. Accounting for an attrition rate of 15 %, we plan to enroll 100 participants in each arm (total study population = 200). We hypothesize that a linguistically tailored health IT intervention based on IVR and SMS will be associated with an improvement in adherence (to anti-platelet and lipid-lowering medications) and an improvement in health literacy in Pakistani patients with vascular disease.

**Discussion:**

This innovative study will provide early data for the feasibility of the use of IT based prescriptions in an lower middle incorme country setting with limited numeracy and literacy skills.

**Trial registration:**

Clinical Trials.gov: NCT02354040 − 2 February 2015

**Electronic supplementary material:**

The online version of this article (doi:10.1186/s13063-016-1244-1) contains supplementary material, which is available to authorized users.

## Background

Vascular risk, manifesting as myocardial infarction (MI) and stroke, contributes to about 3.87 million premature deaths in the 30–69 year-old age group in Pakistan [1]. In one of the largest case-control studies of 15,152 cases of first MI and 14,820 controls, it was shown that South Asian participants were relatively young at the time of their first MI (mean age = 53 years) compared with participants from China or Western Europe (mean age = 63 years) [2]. Nearly 10 % of these heart attacks in South Asian participants occurred in individuals aged 40 or below. Similarly, the reported lifetime prevalence of stroke symptoms is 19 % in urban Pakistan, which amounts to 7 million potential cerebrovascular victims in this densely populated high-risk region [3].

Although most patients admitted with acute coronary syndrome or acute ischemic stroke in South Asian countries receive these evidence-based treatments, their overall continuation in the outpatient phase of care remains low. Patients from Pakistan are uniquely challenged in this respect because the overall literacy rates remain one of the lowest in Pakistan among South Asian countries. In addition, a great majority of Pakistani patients often do not understand or follow health prescriptions (which are still written in English). Additionally, due to an unregulated health industry, patients frequently take multiple opinions and prescriptions from different physicians [1, 4]. This lack of understanding leads to a reduced adherence to these often life-saving medications and increases their risk for drug-drug interactions and serious adverse reactions.

The purpose of this study is to develop and pilot test a tailored health information technology-driven intervention in Pakistani patients with coronary artery disease (CAD) and ischemic strokes receiving care in outpatient setting in the Aga Khan University Hospital (AKUH) in Karachi, Pakistan. We hypothesize that this health IT-driven intervention would address the above-mentioned information gaps and will lead to an increase in medication adherence. Furthermore, we hypothesize that this health IT-driven intervention with improve health literacy in this literacy-challenged, resource-poor population.

## Methods

### Design overview

**Fig. 1:**
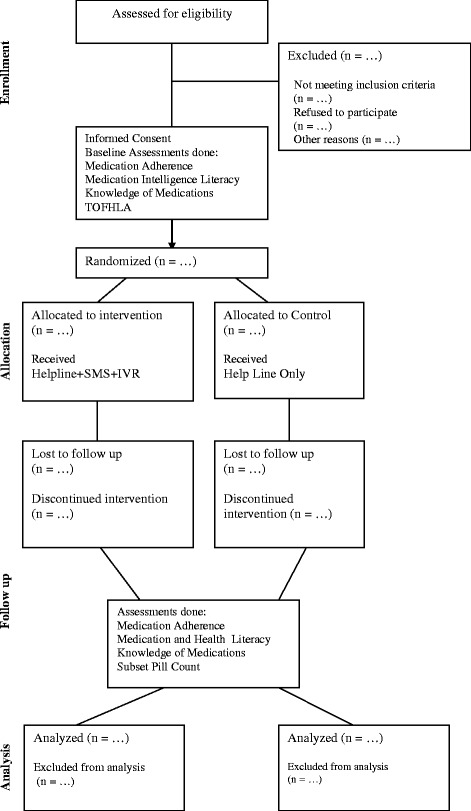
Study flow chart. Standardized Consolidated Standards of Reporting Trials (CONSORT) flow for the Talking Prescription (Talking Rx) program

The *Bolta Parcha* (Talking Prescription; Talking Rx) trial is a randomized, controlled, single center superiority trial with blinded outcome assessment. (Fig. 1 – Study flow chart) Adult participants, with access to a cell phone and a history of vascular disease longer than 1 month of duration, who are taking multiple risk-modifying medications (inclusive of anti-platelets and statins) will be selected from neurology and stroke clinics at the AKUH.

They will be randomized into two parallel groups (intervention group and control group) in a 1:1 ratio via block technique. Both groups will have access to a helpline number to address their queries regarding their illness and medications in addition to receiving the standard of care as per institutional guidelines. In addition, participants in the intervention group will also have access to Interactive Voice Response (IVR)-based informative voice messages tailored to their respective prescriptions. The intervention group will also receive daily tailored text messages regarding dosage and frequency of intake of statins and anti-platelet medications. They would also receive weekly text message reminders to improve medication adherence, lifestyle changes, medication information, risk factors and motivation to improve adherence. These messages are modeled on Social Cognitive Theory and the Health Belief Model and are categorized by Michie’s Taxonomy of Behavioral Change Communication [5, 6].

Patients’ adherence to medications will be assessed using the Morisky Medication Adherence Scale (MMAS) [7]. In a sub-set of patients (20 % of patients in each group), pill count will be performed to assess the correlation between self-reported MMAS and direct pill count. Patient’s health literacy will be measured at baseline and then after 3 months in each group by using Likert’s Scale for Health Intelligence Literacy, Knowledge of Medications, and a modified version of Test of Health Literacy in Adults (TOFHLA) (modified version) [8–10]. The difference in adherence to medication regimen (and health literacy) before and after the intervention will be compared between the two groups to determine the effectiveness of our intervention.

### Study setting

The trial is being conducted in the cardiology and neurology clinics at the AKUH, Karachi, Pakistan. The center is a Joint Commission International Accreditation (JCIA) accredited tertiary care hospital located in Karachi, a large metropolitan city located in the south of Pakistan. The center also has logistic and technical expertise available from dedicated research support staff and an independent Clinical Trials Unit (CTU) and Aga Khan Development Network e-Health Resource Center (AKDN eHRC).

### Participants

Participants attending neurology and cardiology clinics at AKUH are recruited as study participants based on the following criteria:

#### Inclusion criteria

- Age older than 18 years
- History of stroke(s) or CAD (MI, unstable angina, or coronary intervention performed via percutaneous coronary intervention or coronary artery bypass grafting (CABG)) confirmed using objective modalities at the time of the episode (i.e., neuroimaging, electrocardiogram (ECG), relevant blood tests and physician examination and clinical confirmation in medical records)
- More than 1 month since last episode of stroke or admission for CAD as described above
- Use of anti-platelets and statins in addition to other medications to control risk factors of cardiovascular disease
- Modified Rankin Score of 3 or less
- Possession of a personal cell phone that the patient has access to at all times. In the case of patients who do not own, or are unable to use, mobile phones, they must have a caregiver available at all times who possesses a cell phone
- Ability to receive, comprehend and reply to an SMS in English or Urdu or Roman Urdu. In the case of patients who themselves are unable to receive, comprehend or reply to an SMS, they must have caregivers available at all times who could perform the above-mentioned tasks

In addition all participants are required to provide a written, informed consent prior to enrollment.

#### Exclusion criteria

- Intention to travel within 3 months of recruitment
- History of current malignancy (diagnosed in the last 5 years and receiving treatment)
- Planned procedure (in the study time period of 3 months) which necessitates rapid medication changes

### Interventions

#### Intervention group

##### Talking Prescription (*Bolta Parcha*)

**Fig. 2:**
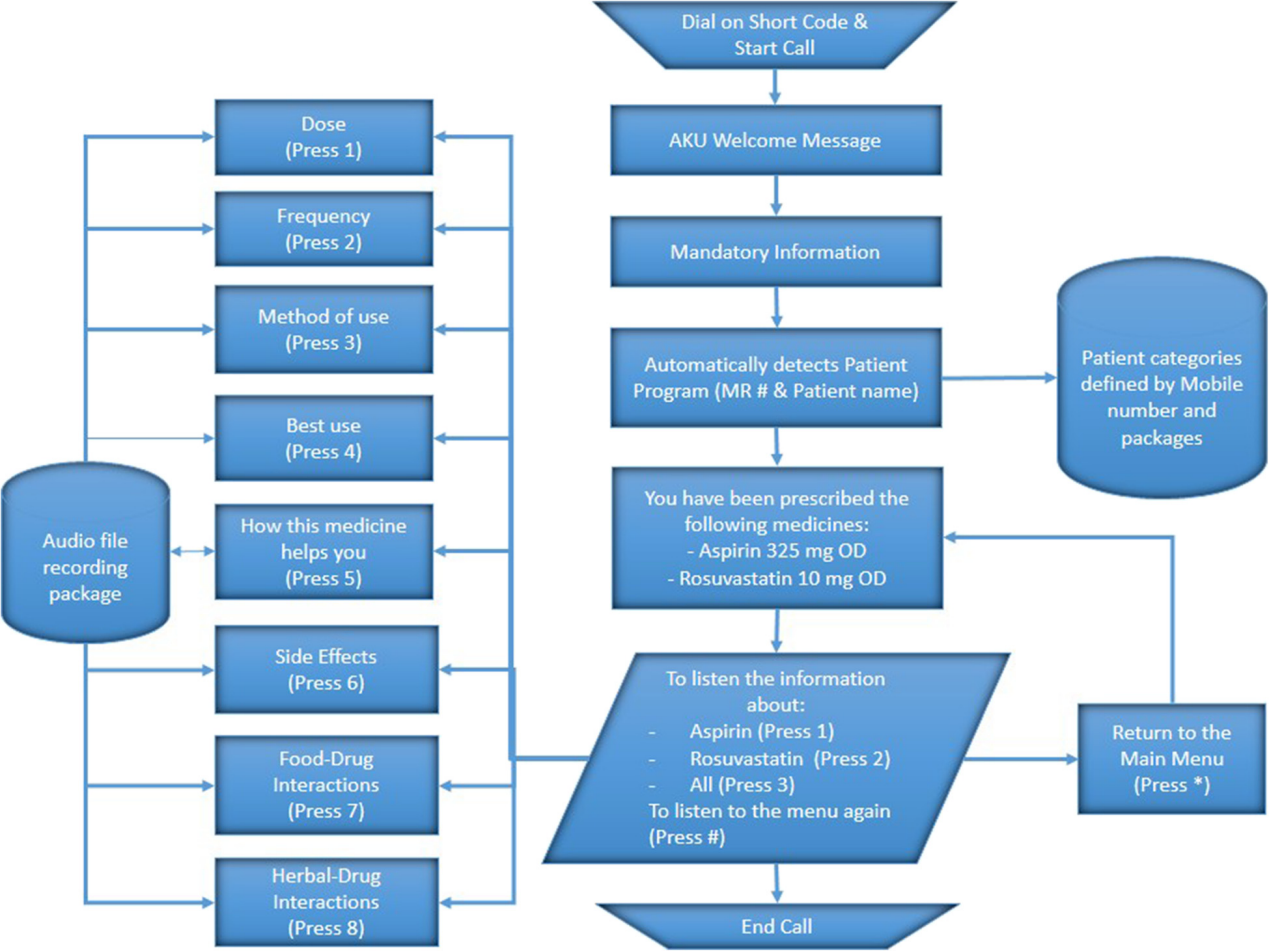
Interactive Voice Recording Menu. Interactive Voice Recording Work Flow (IVR) –the participant is able to “listen to” his medications and how they work, their side effects and important interactions. All participants hear a mandatory recording that details essential information in addition to detailed descriptions. There is no cost to this recording and they may listen to it as many times as they wish for clarity

At enrollment, patients will be allotted unique identities (IDs). Patients in the intervention group will be given access to an IVR which is specific to each patient in terms of the medicines prescribed to that particular patient. (Fig. 2 – Interactive Voice Recording Menu) The physician’s prescription is transferred and scanned and sent to the central program to customize the IVR output for that particular participant. The intervention primarily focuses on two sets of medications (i.e., anti-platelets and statins) used in the treatment of patients with stroke and with CAD. These two medication groups were chosen as they have been shown to improve cardiovascular outcomes in patients with stroke as well as those with CAD.

Patients in the intervention arm will be given a phone number, which they can call on at any time of day or night, where a voice recording will inform the patient that the call is free of charge and they will now receive a call back from AKUH. The patient will then receive a call within a minute from AKUH where an IVR is being played. A mandatory message is played explaining the important instructions pertaining to all medicines. The recording then lists medicines the patient has been prescribed serially and asks patients to choose to receive information about either one medicine or all the medicines in their prescription. If, for example, the patient chooses one medicine, the recording then lists the options available which the patient can choose from (dose, frequency, how and when to take the medicine, drug-drug and drug-food interactions, side effects) and press the assigned digit. The patient can listen to each subcategory serially, or targeted information as desired, once or multiple times. This service is available free-of-cost 24 hours a day, 7 days a week.

This modality stresses the relevant pharmacodynamics and pharmacokinetics explaining all the relevant information to the patient in an effort to maximize the effectiveness of this service. It focuses on educating the patient on how and when to take the prescribed medication and about the necessary storage and usage instructions. It strives to impart detailed information regarding the prescribed anti-platelets and statins to each patient so that medication knowledge, adherence and proper usage are highlighted. It also addresses the common myths that surround medications in health literacy-challenged areas – e.g., the myth that you can feel your chronic disease, and therefore, if you feel better, you can stop taking your medications.

##### Short Text Message Service (SMS)

In addition to the IVR service described above, the participant in the intervention arm will also receive an automated SMS which is highly personalized, tailored to the medication prescription of that particular participant, with emphasis on anti-platelets and statins. Daily automated medication reminder SMS will be sent to all the participants to remind them to take their medicines. Using SMS, the participants in the intervention arm will be asked if they have taken their anti-platelet/statin medication for that day, and if not, then they will be urged to take the medication then.

Furthermore, a SMS will be sent once weekly to each patient in the intervention group which will reinforce the importance of medication adherence, lifestyle modifications and cardiovascular risk factors. These aim to boost medication adherence and health literacy. The SMS are phrased using the behavior change intervention taxonomy devised by Michie et al. [6].

Using the standardized definitions as presented in the taxonomy allows for easy reproducibility and generalized applicability for future work related to this intervention. The specific content of the SMS is derived from the Social Cognitive Theory and Health Belief Model of behavior change in health care [11, 12]. Templates used include “Pakistanis are at high risk of stroke and heart attack. Please reduce your risk by taking your medicines on time” and “Exercise is good for your health. Try to perform regular exercise.” The purpose of adapting this model for designing SMS is to make them effective in communicating to the participants the consequences of their behavior on health.

Please note that both IVR and SMS will be available in English, Roman Urdu or Urdu based on patient preference. (Additional file 1 – SMS messages).

A hosted SMS gateway provided by our trial technical support (eOcean) is used to send the SMS using web services once upon uploading the medicine prescription onto the application server and daily and weekly SMS requested to the same server according to the participant’s prescription and profile [13].

##### Pill count

On a select sub-set of the intervention group (approximately 20 %) medication adherence will be objectively monitored by doing a pill count. Selected patients are provided with two weekly pill organizers each and their anti-platelets and statins are filled in these organizers for 2 weeks. The patient is then asked to return every 2 weeks for a pill count to objectively assess medication adherence as a direct measure of the intervention being provided to these patients via SMS and Talking Prescription. These patients are required to visit the facility for a pill count every 2 weeks until the cessation of the study (i.e., 3 months).

#### Control group

In the control group, patients will receive the usual standard of care provided at AKUH for stroke/CAD patients. This primarily consists of regular follow-up visits (frequency as per patient needs) with their stroke neurologist or cardiologist.

For index stroke patients, the standard schedule for clinic visits after an uncomplicated recovery is 2 weeks, 1 month, 3 months and 6 months. On each clinic visit, a detailed neurological examination is performed, stroke risk factors are assessed and concerns and queries are addressed. Each patient is provided with a telephone number that can be used to reach the stroke team in case of an emergency.

For index CAD patients especially those after a MI the standard outpatient visit schedule after an uncomplicated recovery is at 1 week, 1 month, 3 months and 6 months. On each clinic visit, a detailed cardiovascular examination is performed and CAD risk factors are assessed.

In both clinics, patients will be provided with a helpline number to which they can send a message if they have any queries regarding their illness or medications. These queries will be answered within the next 24 hours by a physician.

In the control group, during clinics, patients are counseled regarding beneficial lifestyle practices and the significance of compliance with medication regimens. They are also are offered reading materials about their disease in English or Urdu. None of these activities, however, are extended beyond their clinic visit. Each patient also receives clinic appointment reminders 1–2 days prior via SMS and/or phone as per the center policy. The control group does not receive any SMS for lifestyle management or an IVR system that describes their medication prescription in detail [14].

The reason for choosing this site and control group is due to the standardization of care at this center and its adherence to protocols. This assures that differences observed are due to the intervention itself and does not allow overestimation of effect.

### Technical detail: Talking Rx intervention

The system operates on the following two types of applications, which are integrated to each other:

1. Desktop-based imaging application: this is used for scanning the prescription page, detecting markers of medications, dose and frequency, and then translating it into an XML form which is understandable by the web-based medical record application
2. Web-based medical record application: this is hosted on a web-based server which registers patients, keeps patients’ pharmacy data, translates XML into record form and communicates with other messaging servers through CSV file and HTTP protocols

In addition to the above-mentioned applications, the system has a messaging and voice server. This server is hosted at the service provider and contains an application which stores all audio files in coded form. The application accepts CSV file format from the web-based server and binds specific audio files and messages based on the prescribed medicines with patients’ mobile phone numbers. This server allows the web-based medical record to send SMS to patients through HTTP protocols so that they can call this server from their mobile phones to hear their prescription.

#### Technical flow

The IVR flow of the intervention is as follows:

1. Patient registration: the patient is registered on web-based application with his/her demographics, history and mobile number
2. Acquisition: after receiving a scanned patient’s prescription on an OMR sheet, a health professional/receptionist/research assistant scans the OMR sheet through a scanner using the web-based application. This web-based application opens a desktop-based application to scan the sheet, convert it into an image and then translate it into XML format
3. Processing: upon scanning, the web-based application obtains an XML file and translates it into patient records on the basis of patient registration number. After obtaining all the information, the web application shows the medical drug status of each patient so that the user can change status, i.e., activate and deactivate a drug accordingly. Upon confirmation of the request, the web-based application sends a message to the messaging and voice server which allocates patient-specific drugs
4. Messaging: upon registration or changing of a prescription, a patient will receive SMS from the messaging server along with an IVR short code to call a specific number to hear their prescription, within 4 hours
5. Voice response: as needed, patients can call the IVR short code at any time and hear their prescription details like frequency, indications, contraindications, side effects, etc.
6. Periodic messaging: based on diseases and drugs, the web-based system sends customized messages to patients through the messaging server periodically. Through this feature, behavior change communication is established to promote positive behavior in patients

### Outcomes: (Additional file 2 − Data Collection Form)

The primary outcome of interest is a change in medication adherence after 3 months of receiving the SMS and being exposed to the Talking Prescription (*Bolta Parcha*). Our secondary outcome is change in health literacy. The findings of the treatment group on these outcomes will be compared to the corresponding findings from the control group to account for the Hawthorne effect.

Medication adherence will be measured at recruitment and after 3 months in both groups using the MMAS. The scale has been used in a similar setting previously and it has been translated and validated in Urdu. The change will be determined by comparing the score on the MMAS before and after the intervention [15].

The instrument consists of eight questions and the response to each question is scored as either 0 (yes) or 1 (no). The maximum score, hence, is 8. A higher score indicates better adherence. A score of less than 2 indicates low adherence.

The secondary outcome measure is assessment of functional health literacy in adults (TOFHLA) which will be assessed at enrollment and then at the follow-up visit after 3 months. This tool has been adapted, modified, and content-validated by a panel of experts, translated and back-translated from English to Urdu, to better represent the Pakistani community and its health system. It consists of two sections: the first section covers questions about the basic instructions for each patient coming to the clinic, and the second section contains three passages comprised of questions about the relevant information every patient visiting the hospital should ideally possess.

Section 1 has a total of 17 questions, where a correct answer merits a score of 1 and an incorrect answer gets a score of 0. First Raw score is obtained after adding responses to each question and this score out of 17 is converted to a weighted score, using a reference table, to a denominator of 50.

Section 2 has a total of 50 questions, where each correct answer earns a score of 1 for the patient and an incorrect answer earns a score of 0. The total score at the end of this section is scored out of 50.

In the end, TOFHLA is evaluated on a total of 100, where health literacy is given a total score ranging between 0 and 59 (inadequate), between 60 and 74 (marginal), and from 75 up to 100 (adequate) [8].

In addition, we will perform sensitivity analyses comparing the correlation between the MMAS and the pill count.

### Study timeline

Prior to each stroke/cardiology clinic visit, potential study participants will be identified from the clinic appointment lists. These patients, along with all other walk-in patients, will be approached by research officers during their visit to assess their eligibility for participation through an eligibility questionnaire. Those found eligible are informed about the study and if willing, will be requested to remain after their appointment for recruitment and providing written, informed consent. If a participant is interested but does not have time, they will be offered information about the study and asked to consider participation in the study at their next clinic visit.

First, counseling is performed by a research officer, in which details of the study, its purpose, objective, procedure and its follow-up visits are explained to the eligible participant. Recruitment will begin with signing of an informed consent and assigning the patient an ID number. Data collection will begin with a detailed interview regarding the demographic and clinical details of the patient including his/her prescription details. The baseline MMAS and TOFHLA scores for each patient will then be assessed. This will be followed by the research staff leaving the room and a senior investigator taking over, at which point the patient will be randomized to either the intervention or the control group by the CTU. The tasks of allocation of intervention and randomization are carried out by the CTU staff (separate from the study staff) and are independent from all studies that are ongoing at the university site.

If the patient is allocated to the intervention arm, the investigator will explain the details of the intervention. The investigator will also demonstrates it by sending one test SMS on his/her cell phone (in English and Urdu both). Any questions pertaining to the use and comprehension of IVR or SMS will be answered at that point.

Patients in the intervention arm will then be provided with a number on which they can dial (free-of-cost) the Talking Prescription. The unblinded investigator will then demonstrate this call to the participant and provide orientation regarding proper use of this IT facility.

If there is a change in patient prescription with respect to any drug or dose by any physician, the patient will be requested to notify this on a helpline number that is provided to them during enrollment. In addition, there is a fortnightly review by a research officer to screen and ensure safety and to draw attention to any prescription change that has not been noticed. This allows the Talking Prescription to be modified so that the patient can then be sent instructions according to the new regimen. The helpline is available round the clock and it could also be reached in the case of any queries. The operator at the helpline is trained to answer and deal with most frequently anticipated questions and queries and is given access to a stroke neurologist and a cardiologist at all times in case of queries beyond the scope of their knowledge. If it is anticipated that the participant will undergo frequent medication changes, associated with, e.g., planned carotid endarterectomy (CEA), CABG, stent, etc., they are not enrolled into the program until their condition is stable.

**Fig. 3:**
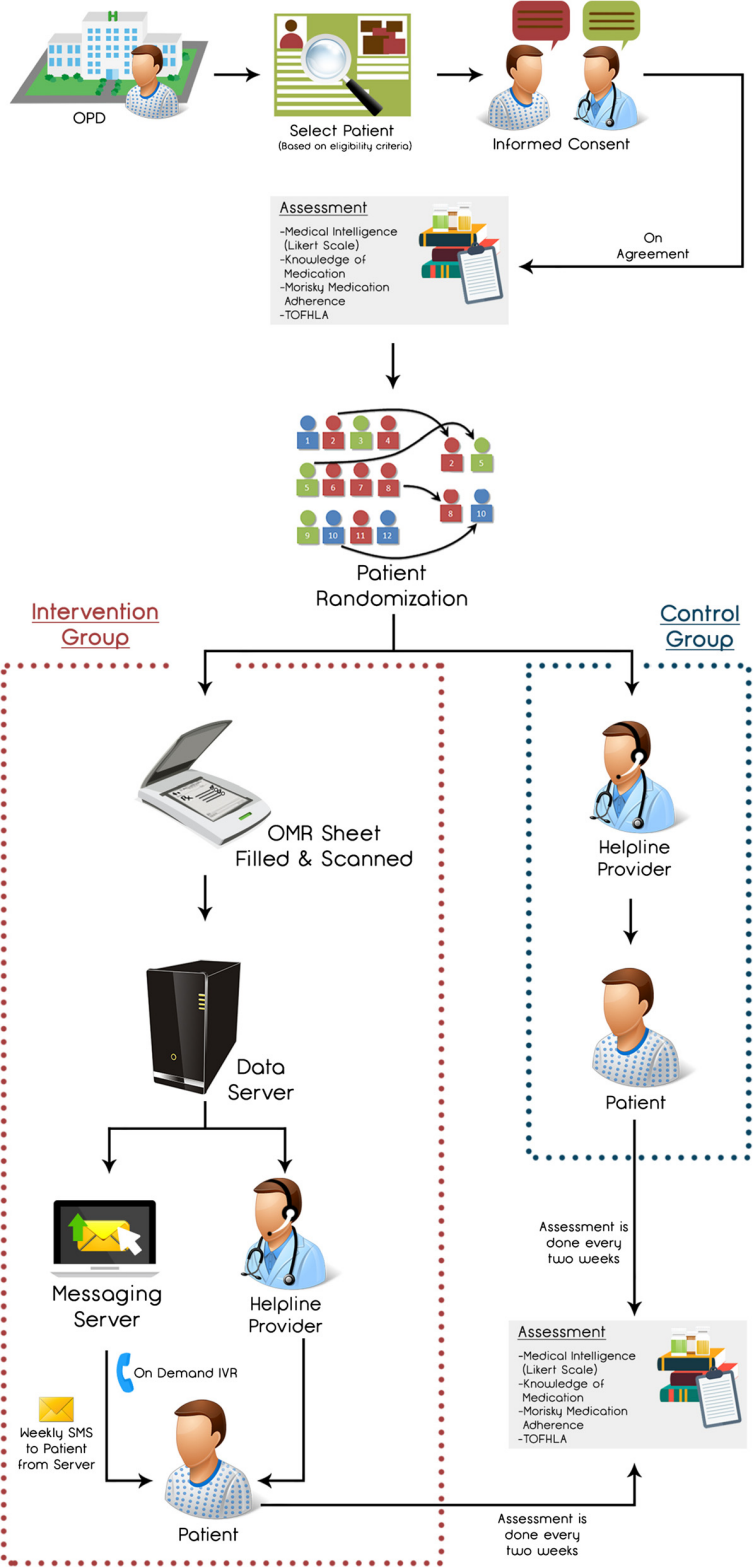
Participant timeline. Timeline for Talking Prescriptions (Talking Rx) − participants are followed for 3 months. The intervention arm receives Interactive Voice Recording (IVR) that improves prescription literacy and a short text message service (SMS) that cues medication adherence behavior. The Pill Count Sub-study is at 2-week follow-up

Following the recruitment visit, the patients will not be required to come back to clinic for any additional visits except the end of the study visit at 3 months. The only exception to this will be the select sub-set which is selected for a pill count, which have a fortnightly review. At the end of the study, patient adherence and health literacy is assessed by the research officer who is unaware of patient allocation. (Fig. 3 − Participant timeline for Talking Rx).

### Duration

The trial will continue until we achieve our sample size of 200 (100 in each arm). Follow-up for each study participant will be 3 months from the day of randomization.

### Sample size

The sample size was calculated to detect a difference of 1 point on the primary outcome measure, MMAS, with a power of 90 % and significance level of 5 % between the two groups. The estimated sample size for the study is 86 participants in each arm. Accounting for a 15 % attrition rate the minimum sample size required for the study is 100 participants per arm, making a total of 200 participants to be randomized. The MMAS categorizes low adherence as a score of <6, medium adherence as a score of 6 to <8, and high adherence as a score of 8 [16]. Any 1-point shift is a clinically important increase in scores. In addition, in a study carried out locally, we have shown the use of IT-based interventions to have effects on this score in these ranges [17, 18].

### Assignment of interventions

Sealed opaque envelopes with patient assignments to either the intervention or the control group will be prepared by the CTU through a computerized block randomization technique in block sizes of 10. This is to ensure similarity between the two groups at all times, permitting interim analysis during the study. Additionally this design block was not discussed with the research team interacting with participant recruitment.

The randomization numbers are sealed in opaque envelopes, marked by ID and kept with the CTU. Even if the envelope was held against bright sunlight, the allocation would remain hidden. After enrollment, obtaining informed consent and completing the baseline measurements, the CTU is informed and randomization of the participant is performed.

### Data collection methods

The patients in both arms will be asked to attend after 3 months (90 days ± 15 days) for a follow-up interview to assess adherence and health literacy. Data will be collected on a data collection form (DCF) by a research officer who is blinded to participant allocation. This primarily consists of the MMAS and modified TOFHLA. In addition, in a select sample of patients, the research officer will also perform pill counts every 2 weeks. (Additional file 2 − Data Collection Form).

Participants in both arms will be reminded of their appointment 2 days prior to the interview. Patients will be compensated for travel to AKUH for the 3-month follow-up visit. In case the participant does not report for this appointment, they will be contacted again to reschedule it.

### Pilot testing

Pilot Testing was performed on 10 % of the sample size, i.e., 20 patients before commencement of the study. As per the actual study, participants were recruited from neurology and cardiology clinics and standard procedures are followed. This allowed testing the intervention for smooth application and any systematic errors. Problems with eligibility criteria were identified and the feasibility of achieving sample size was assessed. The data collection tool was also assessed for flow of questions and comprehensiveness. The intervention was assessed for practicality relating to delivery, accuracy, timeliness, comprehension and satisfaction. The participants in the pilot testing are excluded from final study analysis.

### Data management

Research officers are specifically trained to collect all data during patient interviews leaving no data collection forms incomplete. All data collected will be sent to the research office in sealed envelopes. At the end of each day any data missing from forms will be noted and asked for. In case of missing data, the participant will be contacted to fill the missing information. The data will be entered by a separate team which will enter data on EpiData. Data entry is depersonalized with study ID assignment. This team does not include any members involved with research. As per institutional policy, double entry is mandatory for all data. The data entry operators are blinded to the allocation of each patient. All data entered is backed up centrally.

### Statistical methods

Analysis will be performed using the intention-to-treat principle: an interim analysis after 25 % of the sample has been reached and final analysis after data has been collected from all study participants. Since this is an IT-based intervention, we plan to do an interim analysis to assess technical compliance to intervention, any unexpected side effects like participant fatigue and drop out. We do not expect any serious safety issues to arise as this study only concerns reception of educational intervention.

The use of the intention-to-treat principle for analysis means that all patients will be analyzed in the treatment arm to which they were randomized. Analysis will be performed after the completion of follow-up of all study participants. Protocol deviations and exclusions (with reasons, will be reported for each arm of the trial). Estimates of intervention effects will be presented with 95 % confidence intervals. A two-tailed *P* value of ≤0.05 will be considered statistically significant for the outcome measures.

For descriptive analysis, mean with standard deviation or median with interquartile range will be reported for continuous data. For categorical data, frequency with percentages will be calculated.

The primary outcome analysis will be based on improvement in medication adherence level, assessed by the change in MMAS score at 3 months of follow-up. To report the difference between the mean scores of intervention group and control arm, Student’s *t* test or the Mann-Whitney *U* test will be performed.

To evaluate the secondary outcome, change in functional health literacy assessed by TOFHLA, the difference of proportion between the two arms will be calculated either by Pearson’s chi-square test or Fisher’s exact test.

To evaluate the correlation between adherence to medications and pill counts either Pearson’s correlation or the Spearman rank correlation test will be applied and coefficients will be calculated to report the strength of correlation.

All analysis will be performed on Stata version13.

### Recruitment and retention plan

Patients in the hospital database with diagnoses of stroke or MI are selected, following which their clinical histories are studied in detail to ascertain their eligibility criteria. If a participant is interested in the study, eligibility is assessed during a telephone conversation only. If the patient is found eligible, he or she is given the option of visiting the research facility just for the enrollment interview or to coincide with the interview day with clinical follow-up with the respective consultant at the hospital (in case of an upcoming clinic appointment). In case of difficulty in visiting the facility, free pick up and drop off is offered to patients for the enrollment interview.

In order to retain participants the hotline number is shared for all queries arising during enrollment and participants are thanked for their time during the study. Reminders are sent prior to interview dates and a 2-week margin is given to allow a time suitable for CTU visit. Due to telephone contact we will be able to collect data on reasons for participant discontinuation, loss to follow-up, etc.

### Ethics and human subjects’ protection

All patients taking part in the trial will be required to provide written informed consent at the time of recruitment after thorough counseling by the trial-trained research associate, who is a research associate.

Consent forms have been approved by the Ethical Review Committee (ERC) at AKUH and are available in English and Urdu. The participants will have the right to withdraw at any time during the study. In addition, their inclusion or exclusion from the study will not affect the usual standard care provided to them. Participants will be financially reimbursed for all costs pertaining to their follow-up visits. Participants identified as having depression and dementia will be referred to appropriate physicians for further management (see Additional file 3 – Informed Consent Form).

In addition, special care is taken to maintain patient confidentiality. Health promotional texts will be sent once weekly at times that do not inconvenience the patient such as late at night.

In days of high security alerts in the city and announced SMS blackouts, it is planned to send an advanced SMS the night before to the participant so that there would not be any SMS the next day, hopefully avoiding participant anxiety or uncertainty about continuity of care or reception of services.

All team members have received Good Clinical Practice (GCP) training and a face-to-face training, in a 2-day neuroethics course regarding research in the developing world, where all design aspects for this trial were discussed.

The protocol has been approved by the ERC of the Aga Khan University. The study Ethical Review Number is 3165-MED-ERC_14. Any changes to the protocol will need to be approved by the ERC before implementation and reported to the trial registry at https://clinicaltrials.gov.

Six-monthly reviews on the progress of the trial will be conducted by the ERC including inspection of signed informed consent forms and the study will comply with all ongoing audits for local regulatory compliance.

### Access and dissemination policy

Only the collaborating principal investigators and research team will have access to the study results and will be responsible for publishing them in a timely manner. The funding source will not have any special access or privileges regarding results before publication. The ERC will be given access to data if required. Results will be disseminated through scientific journals, conferences and meetings.

## Discussion

This study protocol presents the design of a randomized controlled trial using Talking Prescriptions for improving medication adherence and health literacy in patients with stroke or CAD.

Technology has been used to increase medication adherence to HIV medications, polio vaccination, and most asthma, hypertension and maternal and child care interventions in developed world populations [19–27]. In general, SMS is the most common technology used for these interventions. This is a patient-centered intervention which is friendly, repeatable and accessible and requires no advanced literacy.

There are several unique aspects to this protocol. First, technology has never been used in a unique high-risk vascular disease population living in resource-strapped settings with marginal support and poor health literacy [28–31]. Second, the content used in this intervention are not just simple knowledge transfer texts, the wording and the content has been designed based on the Health Belief Theory and Social Cognitive Model [32, 33]. Third, each behavioral communication message is informed by taxonomy and coded for easy replicability of intervention [6]. Fourth, our study protocol, design and methods are robust by the use of centralized randomization, allocation concealment, blinded assessment, intention-to-treat analysis, and a design to ensure maximum follow-up and minimize attrition [34–36].

The major limitation of this study is the choice of measure of primary outcome, i.e., self-report of medication adherence on the Morisky Medication Adherence Questionnaire [37, 38]. Although this tool has been validated in Urdu in the implementation country, using assessment tools such as biochemical markers or electronic pill boxes may potentially provide more accurate results. This however is not possible because of the complexity of a vascular patient’s prescription; this may include drugs for multiple risk factors and multiple drugs at the same time. Simply opening an electronic pill box registers a dose as administered even if the patient takes only two medicines out of the four he is prescribed for that time, thus an electronic pill box could not capture the data of a complicated regimen. Running biochemical tests for each of these medications would be costly and impractical in a resource- constrained environment and would require repeat hospital visits [39–42]. In any arm, if the design required patients to make repeat visits during intervention and to interact with the caregiver then the act of repeated interaction may then be more responsible for adherence than the IT-based intervention itself. However, we do intend to perform a limited pill count to add robustness and triangulation data to our measures. Another limitation of our study is that we are unable to blind participants to the educational intervention, so those on the intervention arm are receiving SMS and are thus aware that they are on a program to increase literacy and medication compliance. A possible design to account for participant effect due to the lack of blinding could be giving two versions within the intervention where general information may be imparted in one arm and more detailed specific intervention could be imparted to participants in the second intervention arm as compared to the control. At this pilot stage we were unable to plan for two versions in this intervention arm since we were concerned about how this technology would be received and used in this early study.

Another limitation to the study is the application of the literacy tool TOFHLA in a developing country like Pakistan where the literacy rate varies between 28 and 96 %, varying sharply with age groups and regions. Since Karachi is the largest metropolitan city of the country with population of 9.3 million, literacy rate variations in this city are expected. In addition, the health system in Pakistan is very different to the US where this tool was designed. Hence, TOFHLA was modified and adapted to embrace the limitations encountered in our local community while we adhered to the basic architecture of TOFHLA. First, section 1 was modified to encompass our health system replacing insurance coverage with topics more pertinent to our local health structure. Second, passages in section 2 were completely over-hauled and replaced by our local data and advice by regional content experts. In addition, each statement was kept short and focused with just one blank to decrease the confusion that was anticipated with the presence of three to four blanks in the original version. The total numbers of questions in each section were kept the same to ensure accurate scoring as delineated in the original tool. This modified TOFHLA was content-validated by five experts each, from the fields of neurology, cardiology and epidemiology/statistics. After extensive fine-tuning, this tool was translated in Urdu and thereby successfully tested in the pilot. Our Cumulative Validity Index (CVI) scores for the regionally adapted TOFHLA were as follows: for relevance 0.87 and for clarity 0.72, which are excellent.

This study is regionally important. Pakistan, like other emerging low- and middle-income countries, faces non-communicable diseases like stroke and MI as its major health challenge, so a low-cost intervention would have a high impact [1, 4]. The study population is uniquely at high risk and exceptionally vulnerable to these two vascular diseases. Currently, the country has an electronic database with a unique identifier number, thus the potential of reaching masses as an extension is possible in addition to the already vast mobile infrastructure [43]. Since most out-of-pocket, large, unanticipated expenditure on vascular disease would push most Pakistanis into poverty, simple small steps in prevention would help and, even if the expected effect size is modest, we may ultimately have the potential to empower and positively influence many more lives [44–46].

## Trial status

Ongoing.

## Consent to publish statement

For any participant details, images, or videos used in this publication written informed consent has been provided for the purposes of publication.

## Additional files

Additional file 1: Taxonomy of SMS messages. (DOC 119 kb) Additional file 2: Data Collection Form. (DOCX 84 kb) Additional file 3: Informed Consent Form. (DOCX 18 kb)

## Abbreviations

AKUH: Aga Khan University Hospital
CAD: coronary artery disease
CTU: Clinical Trials Unit
IVR: Interactive Voice Recording
JCIA: Joint Commission International Accreditation
MMAS: Morisky Medication Adherence Scale
SMS: short text message
TOFHLA: Test of Functional Health Literacy in Adults
