# Supplementary material for: Using a tailored health information technology- driven intervention to improve health literacy and medication adherence in a Pakistani population with vascular disease (Talking Rx) – study protocol for a randomized controlled trial
Source: Trials. 2016 Mar 5;17:121. doi: 10.1186/s13063-016-1244-1 (PMC4779210; doi:10.1186/s13063-016-1244-1)
Supplement: Additional file 1: — Taxonomy of SMS messages. (DOC 119 kb) [file 13063_2016_1244_MOESM1_ESM.doc]

**SMS Messages and Codes**

1. You can die if you stop taking your medicines! ( 32)

2. Exercise is good for your health. Try to perform regular exercise. (1)

3. Consume oil for cooking food not ghee. (7)

4. Begin exercise at low level and then gradually increase. ( 5)

5. Fruit and vegetables decrease the risk of stroke and heart attack. Include them in your diet. (1,4)

6. Smoking increases the risk of stroke, heart attack and cancer. Don’t take this risk. Protect yourself and your children.(1,2)

7. You are important to your family. Please take your medicine on time.(3)

8. You don’t want to spend the rest of your life in bed. Take your medicines on time. (4)

9. Pakistanis are at high risk of stroke and heart attack. Please reduce your risk by taking your medicines on time. ( 1,2)

10. Remember your drug timings along with your meal times. Then you will not forget to take your medicines. (15)

11. Next time you travel don’t forget to take your medicines with you. (15)

12. People stop taking their medications when they are feeling better. Please don’t stop your medicines yourself. ( 5)

Coding Taxonomy:

| **Behaviour change technique definitions.** | |
| --- | --- |
| 1. | Provide information on consequences of behavior in *general* |
|  | Information about the relationship between the behaviour and its possible or likely consequences *in the general case*, usually based on epidemiological data, and not personalised for the individual (contrast with technique 2). |
| 2. | Provide information on consequences of behaviour *to the individual* |
|  | Information about the *benefits and costs* of action or inaction to the individual or tailored to a relevant group based on that individual's characteristics (i.e. demographics, clinical, behavioural or psychological information). This can include any costs/benefits and not necessarily those related to health, e.g. feelings. |
| 3. | Provide information about others’ approval |
|  | Involves information about what other people think about the target person's behaviour. It clarifies whether others will like, approve or disapprove of what the person is doing or will do. |
|  | NB: Check that any instance does not also involve techniques 1 (Provide information on consequences of behaviour in *general*) or 2 (Provide information on consequences of behaviour*to the individual*) or 4 (Provide normative information about others’ behaviour). |
| 4. | Provide normative information about others’ behavior |
|  | Involves providing information about what other people are *doing* i.e. indicates that a particular behaviour or sequence of behaviours is common or uncommon amongst the population or amongst a specified group – presentation of case studies of a few others is not normative information. |
|  | NB: this concerns other people's actions and is distinct from the provision of information about others’ approval (technique 3 (Provide information about others’ approval)). |
| 5. | Goal setting (behaviour) |
|  | The person is encouraged to make a behavioural resolution (e.g. take more exercise next week). This is directed towards encouraging people to decide to change or maintain change. |
|  | NB: This is distinguished from technique 6 (goal setting – outcome) and 7 (action planning) as it does not involve planning exactly how the behaviour will be done and either when or where the behaviour or action sequence will be performed. Where the text only states that goal setting was used without specifying the detail of action planning involved then this would be an example of this technique (not technique 7 (action planning)). If the text states that ‘goal setting’ was used if it is not clear from the report, if the goal setting was related to behaviour or to other outcomes, technique 6 should be coded. This includes sub-goals or preparatory behaviours and/or specific contexts in which the behaviour will be performed. The behaviour in this technique will be directly related to or be a necessary condition for the target behaviour (e.g. shopping for healthy eating; buying equipment for physical activity). |
|  | NB: check if techniques applied to preparatory behaviours should also be coded as instances of technique 9 (Set graded tasks). |
| 6. | Goal setting (outcome) |
|  | The person is encouraged to set a general goal that can be achieved by behavioural means but is not defined in terms of behaviour (e.g. to reduce blood pressure or lose/maintain weight), as opposed to a goal based on changing behaviour as such. The goal may be an expected consequence of one or more behaviours, but is not a behaviour per se (see also techniques 5 (Goal setting – behaviour) and 7 (Action planning)). This technique may co-occur with technique 5 if goals for both behaviour and other outcomes are set. |
| 7. | Action planning |
|  | Involves detailed planning of what the person will do including, as a minimum, when, in which situation and/or where to act. ‘When’ may describe frequency (such as how many times a day/week or duration (e.g. for how long). The exact content of action plans may or may not be described, in this case code as this technique if it is stated that the behaviour is planned contingent to a specific situation or set of situations even if exact details are not present. |
|  | NB: The terms ‘goal setting’ or ‘action plan’ are not enough to ensure inclusion of this technique unless it is clear that plans involve linking behavioural responses to specific situational cues, when only described as ‘goal setting’ or ‘action plan’ without the above detail it should be regarded as applications of techniques 5 and 6. |
| 8. | Barrier identification/problem solving |
|  | This presumes having formed an initial plan to change behaviour. The person is prompted to think about potential barriers *and* identify the ways of overcoming them. Barriers may include competing goals in specified situations. This may be described as ‘problem solving’. If it is problem solving in relation to the performance of a behaviour, then it counts as an instance of this technique. Examples of barriers may include behavioural, cognitive, emotional, environmental, social and/or physical barriers. |
|  | NB: Closely related to techniques 7 (action planning) and 9 (set graded task), but involves a focus on specific obstacles to performance. It contrasts with technique 35 (relapse prevention/coping planning), which is about maintaining behaviour that has already been changed. |
| 9. | Set graded tasks |
|  | Breaking down the target behaviour into smaller easier to achieve tasks and enabling the person to build on small successes to achieve target behaviour. This may include increments towards target behaviour or incremental increases from baseline behaviour. |
|  | NB: The key difference to technique 7 (Action planning) lies in planning to perform a sequence of preparatory actions (e.g. remembering to take gym kit to work), task components or target behaviours which are in a logical sequence or *increase in difficulty over time* – as opposed to planning ‘if-then’ *contingencies* when/where to perform behaviours. General references to *increasing* physical activity as intervention goal are not instances of this technique. |
| 10. | Prompt review of behavioural goals |
|  | Involves a review or analysis of the extent to which previously set *behavioural* goals (e.g. take more exercise next week) were achieved. In most cases, this will follow previous goal setting (see technique 5, ‘goal setting-behaviour’) and an attempt to act on those goals, followed by a revision or readjustment of goals, and/or means to attain them. |
|  | NB: Check if any instance also involves techniques 6 (goal setting – behaviour), 8 (barrier identification/problem solving), 9 (set graded tasks) or 11 (prompt review of outcome goals). |
| 11. | Prompt review of outcome goals |
|  | Involves a review or analysis of the extent to which previously set *outcome* goals (e.g. to reduce blood pressure or lose/maintain weight) were achieved. In most cases, this will follow previous goal setting (see technique 6, goal setting-outcome’) and an attempt to act on those goals, followed by a revision of goals, and/or means to attain them. |
|  | NB: Check that any instance does not also involve techniques 5 (goal setting – outcome), 8 (barrier identification/problem solving), 9 (set graded tasks) or 10 (prompt review of behavioural goals). |
| 12. | Prompt rewards contingent on effort or progress towards behavior |
|  | Involves the person using praise or rewards *for attempts* at achieving a behavioural goal. This might include efforts made towards achieving the behaviour or progress made in preparatory steps towards the behaviour, but not merely participation in intervention. This can include self-reward. |
|  | NB: This technique is not reinforcement for performing the target behaviour itself, which is an instance of technique 13 (provide rewards contingent on successful behaviour). |
| 13. | Provide rewards contingent on successful behavior |
|  | Reinforcing successful performance of the specific target behaviour. This can include praise and encouragement as well as material rewards but the reward/incentive must be explicitly linked to the achievement of the specific target behaviour i.e. the person receives the reward if they perform the specified behaviour but not if they do not perform the behaviour. This can include self-reward. Provisions of rewards for completing intervention components or materials are not instances of this technique. References to provision of incentives for being more physically active are not instances of this technique unless information about contingency to the performance of the target behaviour is provided. |
|  | NB: Check the distinction between this and techniques 7 (action planning) and 17 (prompt self-monitoring of behavioural outcome) and 19 (provide feedback on performance). |
| 14. | Shaping |
|  | Contingent rewards are first provided for any approximation to the target behaviour e.g. for any increase in physical activity. Then, later, only a more demanding performance, e.g. brisk walking for 10 min on 3 days a week would be rewarded. Thus, this is graded use of contingent rewards over time. |
| 15. | Prompting generalisation of a target behavior |
|  | Once behaviour is performed in a particular situation, the person is encouraged or helped to try it in another situation. The idea is to ensure that the behaviour is not tied to one situation but becomes a more integrated part of the person's life that can be performed at a variety of different times and in a variety of contexts. |
| 16. | Prompt self-monitoring of behavior |
|  | The person is asked to keep a record of specified behaviour(s) as a method for changing behaviour. This should be an explicitly stated intervention component, as opposed to occurring as part of completing measures for research purposes. This could e.g. take the form of a diary or completing a questionnaire about their behaviour, in terms of type, frequency, duration and/or intensity. Check the distinction between this and techniques 17 (prompt self-monitoring of behavioural outcome). |
| 17. | Prompt self-monitoring of behavioural outcome |
|  | The person is asked to keep a record of specified measures expected to be influenced by the behaviour change, e.g. blood pressure, blood glucose, weight loss, physical fitness. |
|  | NB: It must be reported as part of the intervention, rather than only as an outcome measure. Check the distinction between this and techniques 16 (Prompt self-monitoring of behaviour). |
| 18. | Prompting focus on past success |
|  | Involves instructing the person to think about or list previous successes in performing the behaviour (or parts of it). |
|  | NB: This is not just encouragement but a clear focus on the person's past behaviour. It is also not feedback because it refers to behaviour preceded the intervention. |
| 19. | Provide feedback on performance |
|  | This involves providing the participant with data about their own recorded behaviour (e.g. following technique 16 (prompt self-monitoring of behaviour)) or commenting on a person's behavioural performance (e.g. identifying a discrepancy with between behavioural performance and a set goal – see techniques 5 (Goal setting – behaviour) and 7 (action planning) – or a discrepancy between one's own performance in relation to others’ – note this could also involve technique 28 (Facilitate social comparison). |
| 20. | Provide information on *where and when* to perform the behavior |
|  | Involves telling the person about when and where they might be able to perform the behaviour this e.g. tips on places and times participants can access local exercise classes. This can be in either verbal or written form. |
|  | NB: Check whether there are also instances of technique 21 (Provide instruction on how to perform the behaviour). |
| 21. | Provide instruction on how to perform the behaviour |
|  | Involves *telling* the person *how* to perform behaviour or preparatory behaviours, either verbally or in written form. Examples of instructions include; how to use gym equipment (without getting on and showing the participant), instruction on suitable clothing, and tips on how to take action *Showing* a person how to perform a behaviour without verbal instruction would be an instance of technique 22 only. |
|  | NB: Check whether there are also instances of techniques 5, 7, 8, 9 and 22. Instructions to follow a specific diet or programme of exercise without instructions how to perform the behaviours are not included in this definition. Cooking and exercise classes as well as personal trainers and recipes should always be coded as this technique, but may also be coded as 22 (model/demonstrate the behaviour). |
| 22. | Model/Demonstrate the behaviour |
|  | Involves *showing* the person how to perform a behaviour e.g. through physical or visual demonstrations of behavioural performance, in person or remotely. |
|  | NB: This is distinct from just providing instruction (technique 21) because in ‘demonstration’ the person is able to *observe* the behaviour being enacted. This technique and techniques 21 (Provide instruction on how to perform the behaviour) and may be used separately or together. Instructing parents or peers to perform the target behaviour is not an instance of this technique as fidelity would be uncertain. |
| 23. | Teach to use prompts/cues |
|  | The person is taught to identify environmental prompts which can be used to *remind* them to perform the behaviour (or to perform an alternative, incompatible behaviour in the case of behaviours to be reduced). Cues could include times of day, particular contexts or technologies such as mobile phone alerts which prompt them to perform the target behaviour. |
|  | NB: This technique could be used independently or in conjunction with techniques 5 (goal setting - behaviour) and 7 (action planning; see also 24 (environmental restructuring)). |
| 24. | Environmental restructuring |
|  | *The person* is prompted to alter the environment in ways so that it is more *supportive* of the target behaviour e.g. altering cues or reinforcers. For example, they might be asked to lock up or throw away or their high calorie snacks or take their running shoes to work. Interventions in which the interveners directly modify environmental variables (e.g. the way food is displayed in shops, provision of sports facilities) are not covered by this taxonomy and should be coded independently. |
| 25. | Agree behavioural contract |
|  | Must involve written agreement on the performance of an explicitly specified behaviour so that there is a written record of the person's resolution witnessed by another. |
| 26. | Prompt practice |
|  | Prompt the person to rehearse and repeat the behaviour or preparatory behaviours numerous times. Note this will also include parts of the behaviour e.g. refusal skills in relation to unhealthy snacks. This could be described as ‘building habits or routines’ but is still practice so long as the person is prompted to try the behaviour (or parts of it) during the intervention or practice between intervention sessions, e.g. as ‘homework’. |
| 27. | Use of follow-up prompts |
|  | Intervention components are gradually reduced in intensity, duration and frequency over time, e.g. letters or telephone calls instead of face to face and/or provided at longer time intervals. |
| 28. | Facilitate social comparison |
|  | Involves explicitly drawing attention to others’ performance to elicit comparisons. |
|  | NB: The fact the intervention takes place in a group setting, or have been placed in groups on the basis of shared characteristics, does not necessarily mean social comparison is actually taking place. Social support may also be encouraged in such settings and this would then involve technique 29 (plan social support/social change). Group classes may also involve instruction (technique 21 (provide instruction on how to perform the behaviour)) demonstration (technique 22 (model/demonstrate the behaviour)) and practice (technique 26 (prompt practice)). |
| 29. | Plan social support/social change |
|  | Involves prompting the person to plan how to elicit social support from other people to help him/her achieve their target behaviour/outcome. This will include support during interventions e.g. setting up a ‘buddy’ system or other forms of support and following the intervention including support provided by the individuals delivering the intervention, partner, friends and family. |
| 30. | Prompt identification as role model/position advocate |
|  | Involves focusing on how the person may be an example to others and affect their behaviour, e.g. being a good example to children. Also includes providing opportunities for participants to persuade others of the importance of adopting/changing the behaviour, for example, giving a talk or running a peer-led session. |
| 31. | Prompt anticipated regret |
|  | Involves inducing expectations of future regret about the performance or non-performance of a behaviour. This includes focusing on how the person will *feel* in the future and specifically whether they will feel regret or feel sorry that they did or did not take a different course of action. Do not also code instances of this technique as the more generic providing information on consequences (techniques 1 (provide information on consequences of behaviour in *general* and 2 (provide information on consequences of behaviour *to the individual*)). |
| 32. | Fear arousal |
|  | Involves presentation of risk and/or mortality information relevant to the behaviour as emotive images designed to evoke a fearful response (e.g. ‘smoking kills!’ or images of the grim reaper). Do not also code instances of this technique as the more generic providing information on consequences (techniques 1 (provide information on consequences of behaviour in*general*) and 2 (provide information on consequences of behaviour *to the individual*)). |
| 33. | Prompt self talk |
|  | Encourage the person to use talk to themselves (aloud or silently) before and during planned behaviours to encourage, support and maintain action. |
| 34. | Prompt use of imagery |
|  | Teach the person to imagine successfully performing the behaviour or to imagine finding it easy to perform the behaviour, including component or easy versions of the behaviour. Distinct from recalling instances of previous success without imagery (technique 18 (prompting focus on past success)). |
| 35. | Relapse prevention/coping planning |
|  | This relates to planning how to maintain behaviour that has been changed. The person is prompted to identify in advance situations in which the changed behaviour may not be maintained and develop strategies to avoid or manage those situations. Contrast with techniques 7 (action planning) and 8 (barrier identification/problem solving) which are about initiating behaviour change. |
| 36. | Stress management/emotional control training |
|  | This is a set of specific techniques (e.g. progressive relaxation) which do not target the behaviour directly but seek to reduce anxiety and stress to facilitate the performance of the behaviour. It might also include techniques designed to reduce negative emotions or control mood or feelings that may interfere with performance of the behaviour, and/or to increase positive emotions that might help with the performance of the behaviour. |
|  | NB: Check whether there are any instances of technique 8 (barrier identification/problem solving), which includes identifying emotional barriers to performance, in contrast to the current technique, which addresses stress and emotions, whether they have been identified as barriers or not. |
| 37. | Motivational interviewing |
|  | This is a clinical method including a specific set of techniques involving prompting the person to engage in change talk in order to minimise resistance and resolve ambivalence to change (includes motivational counselling). |
|  | NB: Only rate this technique if explicitly referred to by name, not if one identifies specific elements of it, this may happen if you have prior experience with this technique. |
| 38. | Time management |
|  | This includes any technique designed to teach a person how to manage their time in order to make time for the behaviour. These techniques are not directed towards performance of target behaviour but rather seek to facilitate it by freeing up times when it could be performed. |
|  | NB: Only rate this technique if explicitly referred to by name, not if one identifies specific elements of it, this may happen if you have prior experience with this technique. |
| 39. | General communication skills training |
|  | This includes any technique directed at general communication skills but not directed towards a particular behaviour change. Often this may include role play and group work focusing on listening skills or assertive skills. |
|  | NB: Practicing a particular behaviour-specific interpersonal negotiation e.g. refusal skills in relation to cigarettes or alcohol would not be an instance of this technique. |
| 40. | Stimulate anticipation of future rewards |
|  | Create anticipation of future rewards without necessarily reinforcing behaviour throughout the active period of the intervention. Code this technique when participants are told at the onset that they will be rewarded based on behavioural achievement. |

**Reference for Above Coding:**

1. Michie, S., et al., A refined taxonomy of behaviour change techniques to help people change their physical activity and healthy eating behaviours: the CALO-RE taxonomy. Psychol Health, 2011. 26(11): p. 1479-98.
