# Supplementary material for: Using a tailored health information technology- driven intervention to improve health literacy and medication adherence in a Pakistani population with vascular disease (Talking Rx) – study protocol for a randomized controlled trial
Source: Trials. 2016 Mar 5;17:121. doi: 10.1186/s13063-016-1244-1 (PMC4779210; doi:10.1186/s13063-016-1244-1)
Supplement: Additional file 2: — Data Collection Form. (DOCX 84 kb) [file 13063_2016_1244_MOESM2_ESM.docx]

# ELIGIBILITY FORM

**IDENTIFICATION DATA**

1) Serial number of eligibility: ____________________

2) Medical record #:_______________________

3) Name: _________________________________________________________

4) Phone number: _______________________

5) Address: ________________________________________________________

**MEDICAL CHART REVIEW:**

6) Date of Medical Chart review: ____________________

| S.no | Criteria | Yes | No |
| --- | --- | --- | --- |
| 1 | Age >18 yrs. |  |  |
| 2 | Stroke/Coronary Artery Disease more than 1 month old |  |  |
| 3 | Prescribed both anti-platelet and a statin |  |  |
| 4 | Possessing a personal mobile phone and able to communicate with SMS Urdu/English (either themselves or with the help of caregiver) |  |  |
| 5 | Modified Rankin Score <3 |  |  |
| 6 | No intention to travel for next 3 months |  |  |
| 7 | No current history of malignancy |  |  |
| 8 | No planned procedure that necessitates rapid medications changes |  |  |

All criteria should be answered in YES to be eligible for participation in the study

(S.no 5 exempted only if 1 constant primary caretaker in case of patients with Modified Rankin Score>3)

**This patient is eligible to participate in the Interactive Prescription Study**

**Yes No**

If the patient is eligible but refuses to participate please state the reason for

Non-participation: ______________

**Screening Log**

**Study Identification Number: -** ____________________

**Name of the Patient: -**  **_________________________**

**Age of the Patient: -**  __________ yrs.

**Gender of the Patient: -** ______________

**Name of the Care-giver: -** ___________________________

**Screening Date: -**  __ __ / __ __ / __ __

(dd / mm / yy)

**Eligible for the Study Participation:** 1. YES 2. NO

**Date Enrolled, if eligible:** __ __ / __ __ / __ __

(dd / mm / yy)

**Consent Obtained from the patient: -** 1. YES 2. NO

**Consent Obtained from the Care-giver: -** 1. YES 2. NO

**Ineligible for the Study participation and Reasons: -**

____________________________________________________________________________________________________________________________________________________________

**Consent Refused and Why:-**

**____________________________________________________________________________________________________________________________________________________________**

**Source of the patient: -** 1. Direct 2. Referral from other hospital

**QUESTIONNAIRE FOR INTERACTIVE PRESCRIPTION STUDY:**

*Informed consent should be obtained from the participant before the interview. Participant should be given a copy of your IC for their own records.*

Patient’s name (optional) __________________________

I .D. No (Unique ID given to each participant) ___________________

Patient’s address: _____________________________________________________________

Telephone no Residence: ____________________

Mobile no: ____________________________

Alternate Mobile number: _______________

Time since diagnosed with vascular disease: _________________________

Screening and Enrolment Clinic: 1. CVA_________________

2. CAD_________________

Name of interviewer: _______________________

Signature: ___________________

Date: _________________________

Remarks (if any):________________

**QUESTIONNAIRE:**

**SECTION I-A CO-MORBID CONDITIONS**

| S.NO | Question | Responses |
| --- | --- | --- |
| 1. | Hypertension | 1. Yes ____, Duration:_____ months 2. No ____ |
| 2. | Diabetes | 1. Yes ____, Duration:_____ months 2. No _____ |
| 3. | Dyslipidemia | 1. Yes ____, Duration:_____ months 2. No _____ |
| 4. | Depression | 1. Yes ____, Duration:_____ months 2. No _____ |
| **5.** | Atrial Fibrillation | 1. Yes ____, Duration:_____ months 2. No _____ |
| **6.** | Carotid Stenosis | 1. Yes ____, Duration:_____ months 2. No _____ |
| **7.** | Stents | 1. Yes ____, Duration:_____ months 2. No _____ |
| 8**.** | By-pass surgery (CABG) | 1. Yes ____, Duration:_____ months 2. No _____ |
| 9**.** | Valve replacement | 1. Yes ____, Duration:_____ months 2. No _____ |
| 10. | Central Obesity | _______ cm |
| 11. | Cigarette smoking | 1. Yes ____,   Age started:_____ years  If stopped, age: _____ years  Cigarettes/day (currently): ___   1. No _____ |
| 12. | History of tobacco chewing | 1. Yes ____, Duration:_____ months 2. No _____ |
| 13. | Hospital admission | 1. Yes ____, Duration:_____ months 2. No _____ |

**SECTION I-B FAMILY HISTORY**

| S.NO | Question | Responses | |
| --- | --- | --- | --- |
| 1. | Early Sudden Cardiac Death (Female <45, Male <50) | Father | 1.Yes ____  2. No ____ |
|  |  | Mother | 1.Yes ____  2. No ____ |
|  |  | Brother | 1.Yes ____  2. No ____ |
|  |  | Sister | 1.Yes ____  2. No ____ |
|  |  | Uncle/Aunt  (blood-relation) | 1.Yes ____  2. No ____ |
|  |  | Grandparents | 1.Yes ____  2. No ____ |
| 2. | Stroke | Father | 1.Yes ____  2. No ____ |
|  |  | Mother | 1.Yes ____  2. No ____ |
|  |  | Brother | 1.Yes ____  2. No ____ |
|  |  | Sister | 1.Yes ____  2. No ____ |
|  |  | Uncle/Aunt (blood-relation) | 1.Yes ____  2. No ____ |
|  |  | Grandparents | 1.Yes ____  2. No ____ |

**SECTION I-C SOCIAL STRESSORS**

| S.NO | Examples | Responses | Types |
| --- | --- | --- | --- |
| 1. | Death of a loved one | 1.Yes ____  2. No ____ | Acute |
| 2. | Sudden financial crisis | 1.Yes ____  2. No ____ |  |
| 3. | Recent diagnosis of a terminal illness | 1.Yes ____  2. No ____ |  |
| 4. | Relationship issues | 1.Yes ____  2. No ____ |  |
| 5. | Intermittent episodes of depression | 1.Yes ____  2. No ____ | Episodic |
| 6. | Anxiety disorder | 1.Yes ____  2. No ____ |  |
| 7. | Emotional distress | 1.Yes ____  2. No ____ |  |
| 8. | Ceaseless worrying | 1.Yes ____  2. No ____ |  |
| 9. | Dealing with a chronic/terminal illness for self/family | 1.Yes ____  2. No ____ | Chronic |
| 10. | Long-term relationship problems | 1.Yes ____  2. No ____ |  |
| 11. | Financial crisis | 1.Yes ____  2. No ____ |  |
| 12. | Workplace issues | 1.Yes ____  2. No ____ |  |

**SECTION II: MEDICATION INTELLIGENCE LITERACY**

| S.NO. | QUESTIONS | Strongly Disagree (1) | Disagree (2) | Neither Agree nor Disagree (3) | Agree (4) | Strongly Agree (5) | Likarts’ Score |
| --- | --- | --- | --- | --- | --- | --- | --- |
| 1. | Medicines should be kept out of reach of children |  |  |  |  |  |  |
| 2. | Branded medicine is far superior in efficacy as compared to generic/chemical variety |  |  |  |  |  |  |
| 3. | It is permissible to vary the medicine dosage according to the severity of symptoms felt by the patient |  |  |  |  |  |  |
| 4. | It is acceptable to medicate for symptoms based on information that you know |  |  |  |  |  |  |
| 5. | It is okay to not inform the physician regarding self-medications administered |  |  |  |  |  |  |
| 6. | It is vital to adhere to punctuality in medicine administration as much as possible |  |  |  |  |  |  |
| 7. | It is okay to double dose on the next day in case of last missed dose |  |  |  |  |  |  |
| S.NO. | QUESTIONS | Strongly Disagree (1) | Disagree (2) | Neither Agree nor Disagree (3) | Agree (4) | Strongly Agree (5) | Likarts’ Score |
| 8. | It is acceptable to share medicines between friends/family based on your own information |  |  |  |  |  |  |
| 9. | Ayurvedic/herbal medicines are harmless adjuncts to prescribed medicines and have no side effects/drug-to-drug interactions, therefore we do not have to share this information with the physician |  |  |  |  |  |  |
| 10. | If we feel better (no symptoms) we can stop medicines altogether |  |  |  |  |  |  |
| 11. | Side effects of aspirin are heartburn, nausea/vomiting, gastrointestinal bleeding and stomach cramps |  |  |  |  |  |  |
| 12. | Side effects of statins are headache, flushed skin, myalgia, nausea/vomiting, abdominal cramping and skin rash |  |  |  |  |  |  |

**SECTION III: KNOWLEDGE OF MEDICATION**

| S.NO. | QUESTIONS | ALWAYS (1) | OFTEN (2) | SOMETIMES (3) | OCCASIONALLY (4) | NEVER (5) |  |
| --- | --- | --- | --- | --- | --- | --- | --- |
| 1. | How often are appointment slips written in a way that is easy to read and understand? |  |  |  |  |  |  |
| 2. | How often are medical forms written in a way that is easy to read and understand? |  |  |  |  |  |  |
| 3. | How often are medication labels written in a way that is easy to read and understand? |  |  |  |  |  |  |
| 4. | How often are patient educational materials written in a way that is easy to read and understand? |  |  |  |  |  |  |
| 5. | How often are hospital or clinic signs difficult to understand? |  |  |  |  |  |  |
| S.NO. | QUESTIONS | ALWAYS (1) | OFTEN (2) | SOMETIMES (3) | OCCASIONALLY (4) | NEVER (5) |  |
| 6. | How often are directions on medication bottles difficult to understand? |  |  |  |  |  |  |
| 7. | How often do you have difficulty in understanding written information your health care provider (like a doctor, nurse, nurse practitioner) gives you? |  |  |  |  |  |  |
| 8. | How often do you have problems getting to your clinic appointments at the right time because of difficulty understanding written instructions? |  |  |  |  |  |  |
| S.NO. | QUESTIONS | ALWAYS (1) | OFTEN (2) | SOMETIMES (3) | OCCASIONALLY (4) | NEVER (5) |  |
| 9. | How often do you have problems completing medical forms because of difficulty understanding the instructions? |  |  |  |  |  |  |
| 10. | How often are you unsure on how to take your medication(s) correctly because of problems understanding written instructions on the bottle label? |  |  |  |  |  |  |
| 11. | How confident are you filling out medical forms by yourself? |  |  |  |  |  |  |
| 12. | How often do you read storage instructions on a medication bottle and follow them? |  |  |  |  |  |  |
| 13. | How often do you have someone (like a family member, friend, hospital/clinic worker, or caregiver) help you read hospital materials? |  |  |  |  |  |  |
| 14. | How often do you read the expiry date on a medication bottle before use? |  |  |  |  |  |  |

**SECTION IV: DEMOGRAPHIC AND SOCIO-ECONOMIC DETERMINANTS**

| S. No. | QUESTION | RESPONSES | |
| --- | --- | --- | --- |
| 1 | Date of birth | _______(dd)_____(m) ________(yy) | |
| 2 | Age (in yrs) | ___________ | |
| 3 | Gender | 1. Male _____ 2. Female ______ | |
| 4 | Education (Complete years of education) | ___________ | |
| 5 | Marital status | 1. Single _____ 2. Married ______ 3. Divorced ______ 4. Widowed _______ | |
| 6 | Family status | 1. Joint family ______ 2. Nuclear family ________ | |
| 7 | How many house-hold members are there in the house? | ________ | |
| 8 | Employment status | 1. Employed ______ 2. Unemployed _____ 3. Retired ______ 4. Housewife ______ 5. Daily wage ______ 6. Others (specify)­­­­­­­­­­­­­________ | |
| 9 | Occupation (please specify) | ________ | |
| 10 | Monthly family income | PRS= ________ | |
| 11 | Household Assets | 1. Washing machine |  |
|  |  | 1. Colour TV |  |
|  |  | 1. Cable TV |  |
|  |  | 1. LCD |  |
|  |  | 1. Refrigerator |  |
|  |  | 1. Tape recorder |  |
|  |  | 1. Microwave |  |
|  |  | 1. Freezer |  |
|  |  | 1. CD Player |  |
|  |  | 1. Sewing machine |  |
|  |  | 1. Car |  |
|  |  | 1. Personal Computer |  |
|  |  | 1. Bicycle |  |
|  |  | 1. Motor bike |  |
|  |  | 1. Mobile phone |  |
|  |  | 1. Cooking ware |  |
|  |  | 1. Property |  |
|  |  | 1. Air conditioner/split |  |
|  |  | 1. Laptop |  |
| 12 | Model of vehicle (s) | _______ | |
| 13 | Land ownership (if any) in sq. yards | 1. Yes ______, _______ sq. yards 2. No _______ | |

**SECTION V: MORISKY MEDICATION ADHERENCE**

| S.NO. | QUESTIONS | 1. YES | 1. NO |
| --- | --- | --- | --- |
| 1. | Do you sometimes forget to take your anti-platelet/statin? |  |  |
| 2. | Over the past 2 weeks, were there any days that you did not take your anti-platelet/statin? |  |  |
| 3. | Have you ever cut back or stopped taking your medication without telling your doctor because you felt worse when you took it? |  |  |
| 4. | When you travel or leave home, do you sometimes forget to bring your medication? |  |  |
| 5. | Did you take your anti-platelet/statin yesterday? |  |  |
| 6. | When you feel that your health concern is under control, do you sometimes stop taking your medication? |  |  |
| 7. | Taking medication every day is a real inconvenience for some people. Do you ever feel hassled about sticking to your treatment plan? |  |  |
| 8. | How often do you have difficulty remembering to take your anti-platelet/statin? | | |
| NEVER/RARELY (4) | |  | |
| ONCE IN A WHILE (3) | |  | |
| SOMETIMES (2) | |  | |
| USUALLY (1) | |  | |
| ALL THE TIME (0) | |  | |
| 9. TOTAL SCORE | | | |

**SECTION VI: PRESCRIPTION SCREEN SHOT**

Please affix here

**SECTION VIIA: TOFHLA—PROMPTS**

HAND PATIENT PROMPT FOR EACH QUESTION. THEN READ EACH QUESTION, AND RECORD RESPONSES. STOP AT THE END OF 10 MINUTES.

PREFACE EACH QUESTION WITH:

“These are directions you or someone else might be given at the hospital. Please read each direction to yourself. I will ask you some questions about what it means.”

PREFACE SUCCEEDING QUESTIONS WITH:

“Have a look at this one” OR “Here is another direction you might be given.”

PROMPT 1:

N-1

1. (0)

If you take your first tablet at 7:00 am, when should you take the next one?

_____________________________________________________________

N-2

(1) (0)

And the next one after that?

_____________________________________________________________

N-3

(1) (0)

What about the last one for the day, when should you take that one?

_____________________________________________________________

PROMPT 2:

N-4

(1) (0)

Could you take that medicine on July 10, 2015?

_____________________________________________________________

PROMPT 3:

If you began taking your medicine Tuesday, when should you take it next?

N-5

(1) (0)

_____________________________________________________________

What day would you take it after that?

N-6

(1) (0)

_____________________________________________________________

PROMPT 4:

N-7

(1) (0)

If this were your score, would your blood sugar be normal today?

_____________________________________________________________

PROMPT 5:

N-8

(1) (0)

When is your next appointment?

_____________________________________________________________

N-9

(1) (0)

What is the location of the clinic you should go to?

_____________________________________________________________

PROMPT 6:

N-10

(1) (0)

How many pills should you take?

_____________________________________________________________

PROMT 7:

How many times in a day will you take the medicine on January 5?

N-11

(1) (0)

_____________________________________________________________

When is the date of issue?

N-12

(1) (0)

_____________________________________________________________

N-13

(1) (0)

What date will you stop the medications?

_____________________________________________________________

PROMPT 8:

If you eat lunch at 12:00 noon, and you want to take this medication before

N-14

(1) (0)

Lunch, what time should you take it?

_____________________________________________________________

N-15

(1) (0)

If you forgot to take it before lunch, what time should you take it?

_____________________________________________________________

PROMPT 9:

How many times in a year will you visit your doctor?

N-16

(1) (0)

_____________________________________________________________

PROMPT 10:

When will you get the blood test done?

N-17

(1) (0)

_____________________________________________________________

N-18 Total Raw Score

N-19 Weighted Score

COMMENTS

HAND PATIENT THE READING COMPREHENSION PASSAGES TO BE COMPLETED. FOLD BACK THE PAGE OPPOSITE THE TEXT SO THAT THE PATIENT SEES ONLY THE TEXT.

PREFACE THE READING COMPREHENSION EXERCISE WITH:

“Here are some other medical instructions that you or anybody might see around the hospital. These instructions are in sentences that have some of the words missing. Where a word is missing, a blank line is drawn, and 4 possible words that could go in the blank appear just below it. I want you to figure out which of those 4 words should go in the blank, which word makes the sentence make sense. When you think you know which one it is, circle the letter in front of that word, and go on to the next one. When you finish the page, turn the page and keep until you finish all the pages.”

STOP AT THE END OF 12 MINUTES.

PASSAGE A: GENERAL INSTRUCTIONS AND INFORMATION FOR DIAGNOSTIC ANGIOGRAM

PASSAGE B: PATIENT’S BILL OF RIGHTS AND RESPONSIBILITIES

PASSAGE C: INFORMED CONSENT FOR OPERATION OR OTHER PROCEDURE

**SECTION VIIB: TOFHLA--PASSAGES**

**PASSAGE A**

1. Patient should be ________ for 6 hours before the procedure
2. Fasting
3. Drinking
4. Sleeping
5. Eating
6. I must inform the doctor about previous _________________, if any
7. foreign trips
8. reactions to contrast media
9. hotel stays
10. hobbies
11. Pregnant women should inform the staff, _______ the procedure, about their pregnancy.
12. after
13. At the time of
14. During
15. Before
16. ___________ patient’s personal medical records (e.g. x-rays, ultrasounds, MRI, CT scan, Lab results) when you come for the procedure
17. Bring
18. Leave
19. Discard
20. Submit
21. Notify Angio staff if patient has ________ disease e.g. Hepatitis, measles, chicken pox, AIDS
22. Genetic
23. Chronic disease e.g. Hypertension
24. Infectious
25. Vaccination
26. Both groin areas should be clean and ________ before the procedure
27. shaved
28. cut
29. tied
30. dyed
31. Your procedure cannot be done without ________________
32. appointment
33. application
34. permission (consent)
35. form
36. Inform the staff regarding ___________ you are taking for any disease e.g. ASTHMA, ALLERGIES, DIABETES, ISCHEMIC HEART DISEASE, BLOOD PRESSURE etc.
37. allergies
38. medications
39. surgeries
40. co-morbidities
41. It is important to inform my physician about any _______ to food or medications
42. Likes
43. Dislikes
44. Colors
45. allergies
46. ___________ are an important investigation before the procedure
47. bank accounts
48. Blood tests
49. Form
50. Paper

**POST-PROCEDURE INSTRUCTIONS**

1. After the procedure, ____ can only be started once the patient is fully awake
2. analgesics
3. icing
4. diet
5. pressure application
6. Day care patients are required to stay in Radiology/ Cathlab Recovery area for ________ hours.
7. 0
8. 1
9. 3
10. 24
11. After the angiography, patients are not permitted to _________ immediately
12. sleep
13. stand up
14. smile
15. talk on phone
16. After Angiography, a rest of _________ hours is recommended during hospital stay.
17. 0
18. 1
19. 3
20. 24
21. Immediately after discharge from the hospital, do not perform vigorous _________
22. talking
23. laughing
24. television watching
25. exercise
26. After a minimum of ___ hours of angiography, regular activities can be resumed
27. 6
28. 12
29. 24
30. 48

**PASSAGE B**

1. I have a right to get _______ care regardless of age, gender, nationality or financial discrimination.
2. poor
3. partial
4. best
5. minimalistic
6. I will be provided with proper ________ in all aspects of care.
7. guidance
8. clothes
9. shoes
10. hair
11. I will be informed of my ______ in a manner which I can understand.
12. water
13. box
14. painting
15. rights
16. I and my family will receive _________ about my disease.
17. pillow
18. education
19. glass
20. clock
21. I and my next of kin have a right to be informed about the benefits and risks of the proposed ________.
22. box
23. bag
24. pen
25. plan of care
26. My _________ will be maintained during treatment.
27. silence
28. singing
29. privacy
30. pen
31. My ___________ will be protected.
32. confidentiality
33. house
34. clothes
35. shoes
36. I and my family will be involved in all _________ related to my health.
37. neighbors
38. relatives
39. shoes
40. decisions
41. _____________ will be obtained from me or my family before any procedure.
42. Clothes
43. Informed consent
44. Bags
45. Boxes
46. I and my family have a right to seek __________ medical opinion or refuse treatment.
47. no
48. herbal
49. second
50. green
51. I and my family members are responsible to provide __________________ information for the treatment.
52. false
53. half
54. brief
55. complete and accurate
56. We must abide by the hospital _________________.
57. buildings
58. papers
59. telephones
60. rules and regulations
61. We must _________ from the use of violent behavior.
62. refrain
63. continue
64. progress
65. adjust
66. We must not use _________ language.
67. foreign
68. sign
69. abusive
70. gentle
71. We must __________ with all discharge instructions and follow-up appointments.
72. oppose
73. comply
74. stop
75. hang
76. We must consider the __________ of other patients and hospital staff.
77. rights
78. papers
79. cellphones
80. left
81. As this is a teaching hospital, I understand that my care will be supervised by fully qualified medical practitioner (s), with the involvement of authorized ________________
82. neighbors
83. relatives
84. children
85. students/trainees
86. I have the right to agree or refuse to participate in any _____________ projects affecting my treatment.
87. telephone
88. neighborhood
89. research
90. road
91. I understand that _________ is not allowed anywhere in the hospital premises except in designated areas.
92. standing
93. smoking
94. sitting
95. eating
96. I must not bring _________ personal belongings to the hospital.
97. poor
98. valuable
99. broken
100. small

**PASSAGE C**

1. When you sign a consent, your permission is valid for __________
2. 1 day
3. 30 days
4. 100 days
5. 365 days
6. Consent can be given by a Parent/Guardian in case of __________
7. ADULT
8. FATHER
9. grand father
10. MINOR
11. Consent for an unconscious patient or a disabled patient can be signed by ___________
12. Next of kin
13. Self
14. Receptionist
15. Neighbors
16. Before obtaining my permission, the doctor or members of the team will discuss with me the nature, purpose, as well as the benefits of the proposed procedure and the ________ involved
17. drips
18. cannulas
19. risks
20. bandages
21. My permission for the procedure will be obtained by a __________ of the team
22. student
23. doctor
24. nurse
25. receptionist
26. While obtaining my permission, the doctor will also tell me about possible _________modalities and the risks involved
27. ward
28. alternative
29. bandage
30. metal
31. If necessary, my permission for _________________________________ will be sought
32. calling neighbor
33. sharpening pencils
34. making buildings
35. transfusion of blood and blood products
36. I will also be explained the purpose, benefits, risks, ___________ and the alternatives of such transfusion
37. complications
38. speed
39. thickness
40. color
41. I have to inform my doctor of all _____________ I have
42. assets
43. money
44. allergies
45. cars
46. I should inform my physician about _______________________, if any
47. previous reaction to contrast media
48. bank accounts
49. signature
50. box
51. All the information has been given to me in the ___________ that I understand
52. date
53. language
54. numbers
55. box
56. All the information given is ___________ for me to consent to and to authorize the ‘procedure’
57. long
58. tall
59. sufficient
60. small
61. My ____________ concerning my condition and about the ‘procedure’ will be answered
62. curtains
63. drinks
64. food
65. questions
66. I only sign consent when I am ___________ with the information given to me

1. puzzled
2. sad
3. happy
4. satisfied
5. Total Score
6. TOFHLA

TO BE FILLED IN BY INTERVIEWER ONLY

**SECTION VIII:** Enrollment Clinic 1. Neurology 2. Cardiology

**SECTION VIIIA: STROKE HISTORY**

| S.NO | STROKE | RESPONSES | |
| --- | --- | --- | --- |
| 1. | When did you have stroke | _______(dd)_____(m) ________(yy) | |
| 2. | Etiology | 1. Large artery |  |
|  |  | 1. Small vessel |  |
|  |  | 1. Cardio embolic |  |
|  |  | 1. Others (please specify) |  |
| 3. | Type of stroke | 1. Ischemic |  |
|  |  | 1. Hemorrhagic |  |
| 4. | Severity of stroke by NIHSS |  | |

**SECTION VIIIB: CORONARY ARTERY DISEASE HISTORY**

| S.NO. | CORONARY ARTERY DISEASE | RESPONSES | |
| --- | --- | --- | --- |
| 1. | When did you have CAD event | _______(dd)_____(m) ________(yy) | |
| 2. | Diagnosis | 1. Stable Angina |  |
|  |  | 1. Unstable Angina |  |
|  |  | 1. Myocardial Infarction |  |
|  |  | 1. Chronic Ischemic Cardiomyopathy |  |
|  |  | 1. Congestive heart failure |  |
|  |  | 1. Others (please specify) |  |
