# Supplementary material for: Using a tailored health information technology- driven intervention to improve health literacy and medication adherence in a Pakistani population with vascular disease (Talking Rx) – study protocol for a randomized controlled trial
Source: Trials. 2016 Mar 5;17:121. doi: 10.1186/s13063-016-1244-1 (PMC4779210; doi:10.1186/s13063-016-1244-1)
Supplement: Additional file 3: — Informed Consent Form. (DOCX 18 kb) [file 13063_2016_1244_MOESM3_ESM.docx]

**INFORMED CONSENT**

**Study information**

| **Project Information** | |
| --- | --- |
| Project Title: Using a Tailored Health Information Technology Driven Intervention to Improve Health Literacy and Medication Adherence in a Pakistani Population with Vascular Disease | Study ID: |
| ERC Ref No: 3165-MED-ERC-14 | Sponsor: Baylor Medical College Center for Globalization, USA |
| Principal Investigator: Dr Ayeesha Kamran Kamal | Organization: Aga Khan University |
| Location: Cardiology and Neurology Clinics at AKU | Phone: 9221- 3486- 4559 |
| Other Investigators: Saira Bokhari, Cardiology | Organization: Aga Khan University |
| Location; Aga Khan University, Hospital, Karachi | Phone: 9221- 3486-4559 |

**PURPOSE OF THE STUDY:**

You are being asked to participate in a study on stroke and cardiac patients in which we will examine the effect of IT driven health information intervention to improve patients’ health literacy and medication adherence among Pakistani Vascular patients.

**PROCEDURES:**

All the patients enrolled in the study will be required to provide us information related to your health and socio demography. You will also be asked questions related to the disease you suffer from and its management at three months after enrollment in the study. There will be 2 groups in the study i.e. the intervention and control group. The group allocation will be by law of chance.

If you get a chance to be selected in an intervention group, you will receive regular (weekly), individualized text SMS and voice messages about your anti-platelet and statin medications. It includes the dose, route, frequency, indication, contraindication and any special instructions related to the above mentioned categories of medications. You will also be provided a special code which can be used to request advanced information about your medications.

If you are selected in the control group, you will receive conventional health education in the out-patient clinics by the nurses and your physician.

Both groups will be provided with a helpline number on which they can direct their queries regarding their illness and medications via text messages. Their concerned consultants will answer their respective queries via text messages within the next 24 hours.

A small sub-section of the participants (chosen randomly) will be provided with pill organizers whereby pill count will be done in this particular group every 2 weeks until the termination of this study (i.e. 3 months).

**RISKS OR DISCOMFORT**

The study does not impose any risk or discomfort to any patient or their caregiver. You will have to come for the follow-up visit at the scheduled time set at the time of enrollment in the study. Thus it is expected that you will have to make time for the follow up visits.

**POSSIBLE BENEFITS:**

You will receive education about your blood thinners and cholesterol medications, free of cost that will improve your knowledge and skills to manage your illness at home and prevent from potential compilations.

**FINANCIAL CONSIDERATIONS**

The cost of your transportation for the follow-ups will be funded by the study, regardless of your assignment, whether you get the informational service or not.

**AVAILABLE TREATMENT ALTERNATIVES**

The alternative source of health education is the conventional form of health education provided by the physicians and nurses which are inadequate to develop behavioral changes among the patients.

**AVAILABLE MEDICAL TREATMENT FOR ADVERSE EXPERIENCES**

It is not expected that you will incur a medical treatment as a result of IT driven health intervention. However since you are our study participant, we will assist you in directing you to your correct care provider should an unrelated medical issue arise during the course of your study. Additionally, your participation in this study will not affect the care and follow up that you will continue to receive in the clinic.

**CONFIDENTIALITY**

Your identity in this study will be kept confidential and the results of the study will not disclose your identity. Your details will be given unique identity numbers or codes to maintain confidentiality. However any records or data obtained as a result of your participation in this study may be inspected by the sponsor or the AKU ERC members.

**TERMINATION OF RESEARCH STUDY**

The results of the study will be communicated to you. You are free to choose whether or not to participate in this study. There will be no penalty or loss of benefits to which you are otherwise entitled in this hospital. In case of your discontinuation of participation please notify Study Team at 0341-2241334 of your decision at the provided telephone number so that your participation can be terminated.

**AVAILABLE SOURCES OF INFORMATION**

Any further questions you have about this study will be answered by the Principal Investigator: Dr Ayeesha Kamran Kamal.

**AUTHORIZATION**

I have read and understand this consent form, and I volunteer to participate in this research study. I understand that I will receive a copy of this form. I voluntarily choose to participate, but I understand that my consent does not take away any legal rights in the case of negligence or other legal fault of anyone who is involved in this study.

Name of participant (Printed or Typed):

Date:

Signature of participant:

Date:

Signature of Principal Investigator:

Date:

Signature of person obtaining consent:

Date:
